# Zymographic differentiation of [NiFe]-Hydrogenases 1, 2 and 3 of *Escherichia coli* K-12

**DOI:** 10.1186/1471-2180-12-134

**Published:** 2012-07-06

**Authors:** Constanze Pinske, Monique Jaroschinsky, Frank Sargent, Gary Sawers

**Affiliations:** 1 Division of Molecular Microbiology, University of Dundee, College of Life Sciences Dundee DD1 5EH, Scotland, UK; 2 Institute for Biology/Microbiology, Martin-Luther University Halle-Wittenberg, Kurt-Mothes-Str. 3, 06120, Halle (Saale), Germany; 3 Molecular Microbiology, College of Life Sciences, University of Dundee, Dow Street, DD1 5EH, Dundee, United Kingdom

**Keywords:** NiFe, Hydrogenase, Formate hydrogenlyase, Formate dehydrogenase, Non-denaturating polyacrylamide gel electrophoresis, In-gel activity staining, Redox-dyes

## Abstract

**Background:**

When grown under anaerobic conditions, *Escherichia coli* K-12 is able to synthesize three active [NiFe]-hydrogenases (Hyd1-3). Two of these hydrogenases are respiratory enzymes catalysing hydrogen oxidation, whereby Hyd-1 is oxygen-tolerant and Hyd-2 is considered a standard oxygen-sensitive hydrogenase. Hyd-3, together with formate dehydrogenase H (Fdh-H), forms the formate hydrogenlyase (FHL) complex, which is responsible for H_2_ evolution by intact cells. Hydrogen oxidation activity can be assayed for all three hydrogenases using benzyl viologen (BV; *E*_o_′ = -360 mV) as an artificial electron acceptor; however ascribing activities to specific isoenzymes is not trivial. Previously, an in-gel assay could differentiate Hyd-1 and Hyd-2, while Hyd-3 had long been considered too unstable to be visualized on such native gels. This study identifies conditions allowing differentiation of all three enzymes using simple in-gel zymographic assays.

**Results:**

Using a modified in-gel assay hydrogen-dependent BV reduction catalyzed by Hyd-3 has been described for the first time. High hydrogen concentrations facilitated visualization of Hyd-3 activity. The activity was membrane-associated and although not essential for visualization of Hyd-3, the activity was maximal in the presence of a functional Fdh-H enzyme. Furthermore, through the use of nitroblue tetrazolium (NBT; *E*_o_′ = -80 mV) it was demonstrated that Hyd-1 reduces this redox dye in a hydrogen-dependent manner, while neither Hyd-2 nor Hyd-3 could couple hydrogen oxidation to NBT reduction. Hydrogen-dependent reduction of NBT was also catalysed by an oxygen-sensitive variant of Hyd-1 that had a supernumerary cysteine residue at position 19 of the small subunit substituted for glycine. This finding suggests that tolerance toward oxygen is not the main determinant that governs electron donation to more redox-positive electron acceptors such as NBT.

**Conclusions:**

The utilization of particular electron acceptors at different hydrogen concentrations and redox potentials correlates with the known physiological functions of the respective hydrogenase. The ability to rapidly distinguish between oxygen-tolerant and standard [NiFe]-hydrogenases provides a facile new screen for the discovery of novel enzymes. A reliable assay for Hyd-3 will reinvigorate studies on the characterisation of the hydrogen-evolving FHL complex.

## Background

Under anaerobic conditions *Escherichia coli* synthesizes three membrane-associated [NiFe]-hydrogenases (Hyd), although its genome has the capacity to encode four of these enzymes [1,2]. Hyd-1 and Hyd-2 are respiratory hydrogenases with their active sites facing the periplasm and the structural subunits of these are encoded within the *hya* and *hyb* operons [3,4], respectively. The physiological role of both enzymes is to couple hydrogen oxidation to the reduction of the quinone pool in the inner membrane, and they can be readily isolated and characterised in an active form [5-8]. Hyd-1 is an oxygen-tolerant hydrogenase while Hyd-2 is a ‘standard’ oxygen-sensitive enzyme [8] and it has been proposed that Hyd-1 functions at more positive redox potentials, which are found at the aerobic-anaerobic interface [8-10].

Hyd-3 is encoded by the *hyc* operon [11,12] and forms a key component of the formate hydrogenlyase (FHL) complex, which is predicted to be associated with the cytoplasmic side of the inner membrane and catalyses hydrogen and carbon dioxide production from formate. Expression of FHL is maximal under fermentative conditions in the absence of exogenous electron acceptors and is absolutely dependent on formate [13]. Hyd-3 is considered a labile hydrogenase that has so far proven recalcitrant to isolation in an active form [14]. The labile molybdenum- and selenium-dependent formate dehydrogenase-H (Fdh-H) is also associated with the FHL complex [15]. Fdh-H represents one of the three formate dehydrogenase enzymes in *E. coli* (Fdh-H, Fdh-O, and Fdh-N) [16]. Fdh-O and Fdh-N are membrane-bound and periplasmically-oriented respiratory enzymes that couple formate oxidation to quinone reduction and thus contribute directly to energy conservation.

Several methods have been described for visualizing the redox activity of hydrogenases. Most commonly, low-potential artificial redox-active viologen dyes such as methyl viologen (MV) and benzyl viologen (BV) have been used [17,18]. All three *E. coli* hydrogenases can couple H_2_ oxidation to BV reduction *in vitro* and when extracts from fermentatively-grown cells are assayed Hyd-3 can contribute over 90% to the total activity [19,20]. While Hyd-1- and Hyd-2-catalysed BV reduction can be readily visualised and the enzymes distinguished by use of an in-gel assay [18], Hyd-3 activity has so far proved recalcitrant to zymographic identification and this had been thought to be due to the instability of the large FHL complex (see [1]). Moreover, the large respiratory Fdh-N and Fdh-O enzyme complexes also contribute some background staining due to their inherent H_2_:BV oxidoreductase activities, thus making any assessment of a Hyd-3 associated activity potentially problematic [21]. Alternative hydrogenase assays have been developed for other biological systems. For example, the oxygen-tolerant hydrogenases from *Ralstonia eutropha* H16 can be visualized with phenazine methosulfate (PMS)/nitroblue tetrazolium (NBT) [22] or PMS/triphenyl tetrazolium chloride (TTC) [23] combinations of redox dyes. Methylene blue has also been used extensively in hydrogenase research [24]. However, the use of alternative redox-active electron acceptors has not really been extensively explored for the hydrogenases of *E. coli*.

The aim of this study, therefore, was to investigate the differential activities of the *E. coli* hydrogenases with a view to making it possible to distinguish all enzymes synthesized under anaerobic growth conditions. We describe here conditions that allow the unequivocal visualization of all three, membrane-associated, anaerobically inducible hydrogenase enzyme complexes.

## Results

### Identification of Hyd-3 activity through an in-gel assay

**Figure 1:**
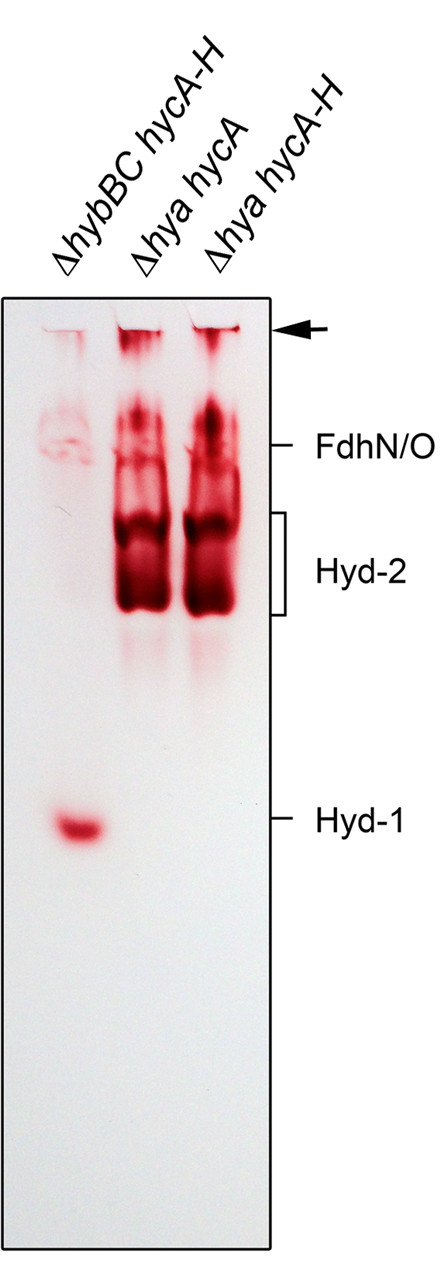
**Identification of hydrogenases 1 and 2 in defined hydrogen metabolism mutants.** Extracts from strains HDK203 (Δ*hybBC hycA-H*), which is Hyd-1^+^, HDK101 (Δ*hya hycA*), which is Hyd-2^+^ and Hyd-3^+^ and HDK103 (Δ*hya hycA-H*), which is Hyd-2^+^ were derived from cells after anaerobic growth in TGYEP, pH 6.5 and 25 μg of protein were applied to non-denaturating PAGE (7.5% w/v polyacrylamide). After electrophoresis the gel was stained in an anaerobic glove box in the presence of ≤5% H_2_ with BV and TTC as described in the Methods section. On the right hand side of the figure the migration patterns of the formate dehydrogenases N and O (Fdh-N/O) and the hydrogenases (Hyd) 1 and 2 are given. The top of the gel is marked by an arrow.

Hyd-1 and Hyd-2 are readily visualized after gel electrophoresis under non-denaturing conditions in a high-pH buffering system [18-20]. Through the use of defined hydrogenase structural gene mutants it is possible to identify which hydrogenase enzyme is responsible for which activity band and this is exemplified in Figure 1. Hyd-1 migrates as a single, fast-migrating activity band and introduction of a mutation in the *hyaB* gene, encoding the large subunit, abolished activity (Figure 1). Hyd-2, on the other hand, migrates as two more slowly-migrating activity bands and these are no longer detectable in *hybC* deletion mutant (Figure 1; [20]). Through the analysis of defined mutants lacking all 3 hydrogenases, it has been shown recently that the respiratory Fdh-N and Fdh-O enzymes also exhibit a H_2_:BV oxidoreductase activity, thus potentially defining a new class of hydrogenase [21]. The weak hydrogenase activity due to Fdh-N and Fdh-O is clearly visible in a crude extract derived from strain HDK203, which lacks functional Hyd-2 and Hyd-3 enzymes (left lane of Figure 1). No other H_2_:BV oxidoreductase enzyme activity is discernible under the conditions used in the experiment shown in Figure 1.

**Figure 2:**
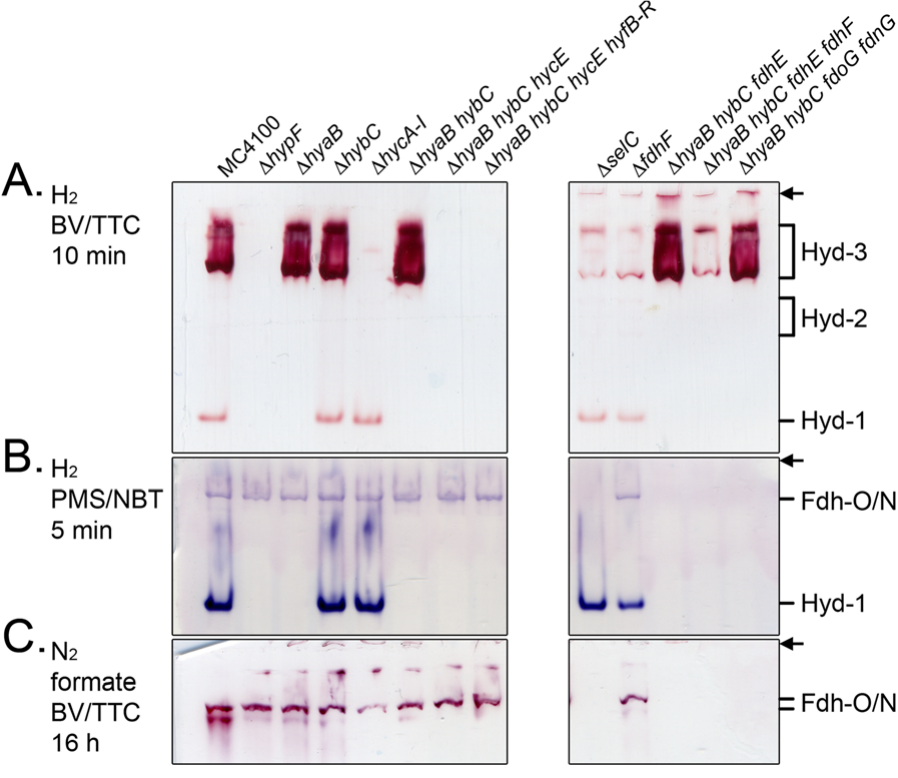
**Staining comparison using hydrogen or formate as electron donor and different redox dye acceptors identifies Hyd-3 activity.** Extracts from the strains MC4100, DHP-F2 (Δ*hypF*), FTD22 (Δ*hyaB*), FTD67 (Δ*hybC*), CP971 (Δ*hycA-I*), CP734 (Δ*hyaB hybC*), FTD147 (Δ*hyaB hybB hycE*), FTD150 (Δ*hyaB hybC hycE hyfB-R*), FM460 (Δ*selC*), FM911 (Δ*fdhF*), CPD17 (Δ*hyaB hybC fdhE*), CPD23 (Δ*hyaB hybC fdhE fdhF*) and CPD24 (Δ*hyaB hybC fdoG fdnG*) that were grown anaerobically in TGYEP media, pH 6.5 were used and 25 μg of protein were applied to non-denaturating PAGE (7.5% w/v polyacrylamide) and stained as indicated with either **A**: BV and TTC under a 100% hydrogen atmosphere, **B**: PMS and NBT under a 100% hydrogen atmosphere, or with **C**: BV, TTC and formate under 100% nitrogen atmosphere. In the interest of clarity only the genotypes of the strains are given. On the right hand side of the figure the migration patterns of hydrogenase 1 (Hyd-1), Hyd-2 and the mixed species of Fdh-N and Fdh-O (Fdh-N/O) are indicated, as well as the presumed migration of active FHL (Hyd-3). The top of each gel is marked by an arrow.

The conditions under which activity-staining is normally carried out involve long incubation times and a gas atmosphere of ≥ 95% nitrogen/≤ 5% hydrogen [20]. Because the Hyd-3 enzyme component of the FHL complex normally catalyzes proton reduction rather than hydrogen oxidation *in vivo* and the spectrophotometric assay of this enzyme typically involves using saturating hydrogen concentrations, and consequently a very low redox potential in the assay, we decided to perform an in-gel activity stain under a 100% hydrogen gas atmosphere. Surprisingly, after exposure for only 10 minutes (see Methods) a prominent and highly active, high molecular weight complex showing H_2_:BV oxidoreductase activity appeared when the native gel was incubated in the presence of a 100% hydrogen atmosphere (Figure 2A, left panel). Although active Hyd-1 could also be detected, no activity bands corresponding to either Hyd-2 or the Fdh-N/O enzymes were observed under these conditions. The activity of this high-molecular weight complex was shown to be dependent on the presence of the *hyc* genes, as it was absent in extracts of strains CP971 (Δ*hycA-I*), FTD147 (Δ*hyaB hybC hycE*) and FTD150 (Δ*hyaB hybC hycE hyfB-R*) (Figure 2A). These data suggest strongly that the high molecular weight hydrogenase activity band corresponds minimally to the Hyd-3 component of the FHL complex, and perhaps even to the intact FHL complex. As mentioned above, it is well documented that Hyd-3 catalyzes hydrogen oxidation *in vitro* and can contribute ~ 90% of total hydrogen oxidation activity measured in crude extracts derived from fermentatively-grown cells [19,20].

### Fdh-H is required to stabilize Hyd-3 but is not essential for activity

Because the FHL complex comprises not only Hyd-3 but also Fdh-H, it was necessary to determine whether the Fdh-H component was required for the visualization of the Hyd-3 activity. Analysis of extracts derived from strains devoid either of the respiratory formate dehydrogenases, Fdh-O and Fdh-N, (CPD24 *hyaB hybC fdoG fdnG*), or the biosynthetic accessory protein FdhE involved in their assembly (CPD17 *hyaB hybC fdhE*) [25,26], clearly showed that the Hyd-3 activity band had similar intensity to that in the wild-type (Figure 2A, right panel). However, when the *fdhF* gene encoding Fdh-H was deleted either alone (FM911), or in combination with *fdhE* (CPD23), the intensity of the Hyd-3 activity band was significantly reduced (Figure 2A, right panel). A similar result was observed when a crude extract derived from the *selC* mutant FM460, which cannot synthesize selenoproteins [27], was analysed. If membrane-associated, it would be expected that Fdh-H migrates together with Hyd-3 as part of a large FHL complex. In-gel formate-dependent BV reduction was therefore tested with the same samples of crude extracts. Following 16 h incubation with formate and BV/TTC under a N_2_ atmosphere two bands showing formate:BV oxidoreductase activity were observed, which migrated slightly more slowly that the Hyd-3 activity and with a much sharper banding pattern (Figure 2B). However, as these activity bands were clearly visible in an *fdhF* deletion strain (FM911), they could not be attributable to Fdh-H (Figure 2B right panel). Rather, the fact that they were absent in extracts derived from FM460 (Δ*selC*), mutants CPD17 and CPD23 (see Table 1) both devoid of *fdhE*, and mutant CPD24 unable to synthesize the Fdh-N and Fdh-O enzymes, this indicates that these activities were due to the respiratory formate dehydrogenases (Figure 2B, right panel). Taken together, these findings indicate that Fdh-H does not appear to co-migrate with Hyd-3 in an enzymically active form. Despite the fact that the Fdh-H component of the FHL complex does not appear to be associated with the Hyd-3 enzyme complex after electrophoretic separation in the gel system used and is not absolutely essential for visualization of Hyd-3 activity, it nevertheless appears to be required to stabilize the active complex.

**Table 1:** Strains and references

**Strain**	**Genotype**	**Reference**
MC4100	F^-^, *araD139,* Δ(*argF-lac*)*U169,* λ^-^, *rpsL150, relA1 deoC1, flhD5301*, Δ(*fruK-yeiR*)*725*(*fruA25*), *rbsR22*, Δ(*fimB-fimE*)*632*(::IS*1*)	[28]
CP734	MC4100 Δ*hyaB hybC*	[20]
CP971	MC4100 Δ*hycA-I*	[29]
CPD17	MC4100 Δ*hyaB hybC fdhE*	This study
CPD23	MC4100 Δ*hyaB hybC fdhE fdhF* (Km^R^)	This study
CPD24	MC4100 Δ*hyaB hybC fdoG fdnG* (Km^R^)	This study
DHP-F2	MC4100 Δ*hypF*	[30]
FM460	MC4100 Δ(*selC*)*400* (Km^R^)	[27]
FM911	MC4100 Δ*fdhF recA56*	[31]
FTD22	MC4100 Δ*hyaB*	[32]
FTD67	MC4100 Δ*hybC*	[32]
FTD147	MC4100 Δ*hyaB* Δ*hybC* Δ*hycE*	[33]
FTD150	MC4100 Δ*hyaB* Δ*hybC* Δ*hycE* Δ*hyfB-R*	[33]
FTH004	MC4100 coding for a chromosomal in-frame *C*-terminal His-tag on HyaA	[34]
HDK101	MC4100 Δ*hya* (Km^R^) Δ*hycA*	Martin Sauter
HDK103	MC4100 Δ*hya* (Km^R^) Δ*hycA-H*	[35]
HDK203	MC4100 Δ*hybBC* (Km^R^) Δ*hycA-H*	[35]
ML23	FTH004 encoding C19G/C120G exchange in HyaA	[9]
ML24	FTH004 encoding a C120G exchange in HyaA	[9]
ML25	FTH004 encoding a C19G exchange in HyaA	[9]

### The large Hyd-3 protein complex is active in a neutral pH gel-system and is membrane-associated

**Figure 3:**
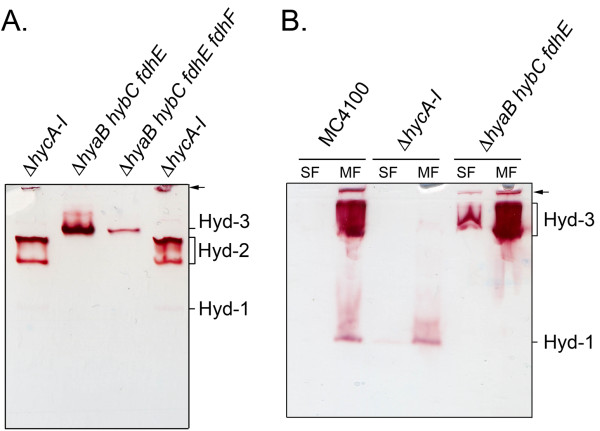
**Hyd-3 activity is detectable after electrophoresis in different gel-systems.** The strains CP971 (Δ*hycA-I*), CPD17 (Δ*hyaB hybC fdhE*), CPD23 (Δ*hyaB hybC fdhE fdhF*) and MC4100 were grown anaerobically in TGYEP, pH 6.5. **A**: About 25 μg of total protein were applied to a Tris-barbitone gel system, pH 7.0 (7.5% w/v polyacrylamide) and the gel was stained in 100% hydrogen with BV/TTC after electrophoresis. **B**: Extracts of the given strains were separated into soluble fraction (SF) and membrane fraction (MF) by ultracentrifugation and 25 μg of each fraction were applied to native PAGE (7.5% w/v polyacrylamide in Tris/glycine system). On the right hand side of the figures the top of the gel is marked with an arrow and the migration patterns of hydrogenase 1 (Hyd-1), Hyd-2 and Hyd-3 are indicated.

The total hydrogen-oxidizing activity measureable in crude extracts of fermentatively grown *E. coli* cells is stable over a broad range of pH but above pH 9 the activity is rapidly lost [18]. To determine whether Hyd-3 activity is detectable also after electrophoresis in a neutral pH buffer system, crude extracts of the strains CP971 (Δ*hycA-I*), CPD17 (Δ*hyaB hybC fdhE*) and CPD23 (Δ*hyaB hybC fdhE fdhF*) were analysed in a Tris-barbitone pH 7 buffer system [18]. The activity of Hyd-3 could be clearly observed as a single, large, slowly-migrating complex (Figure 3A). Once again, while the Fdh-H component was not absolutely essential for activity to be observed, Hyd-3 activity was significantly reduced in a mutant unable to synthesize the enzyme. It was noted that in the neutral pH buffer system the intensity of the Hyd-2 activity bands was much higher after exposure to hydrogen for 10 min than at high pH where it was not detectable in this time-frame (compare Figures 2A and 3A). This is probably due to the fact that Hyd-2 is slowly inactivated by exposure to high pH buffer [5,18]. Hyd-1 activity, in contrast, showed the opposite effect of being more active at high pH and less active in the neutral pH gel-system.

The FHL complex is associated with the cytoplasmic membrane and the active site of each enzyme component (Fdh-H and Hyd-3) faces the cytoplasm [1]. To determine whether the Hyd-3 activity identified in this study was membrane-associated the crude extracts derived from anaerobically grown wild-type (MC4100), CP971 (Δ*hycA-I*) and CPD17 (Δ*hyaB hybC fdhE*) were separated into soluble and membrane fractions and an aliquot of each was separated in the high-pH gel-system and stained for Hyd-3 activity in an atmosphere of 100% hydrogen (Figure 3B). The results clearly demonstrate that Hyd-3 activity, along with that attributable to Hyd-1, was membrane-associated.

### High hydrogen partial pressure facilitates detection of Hyd-3 activity after native-PAGE

**Figure 4:**
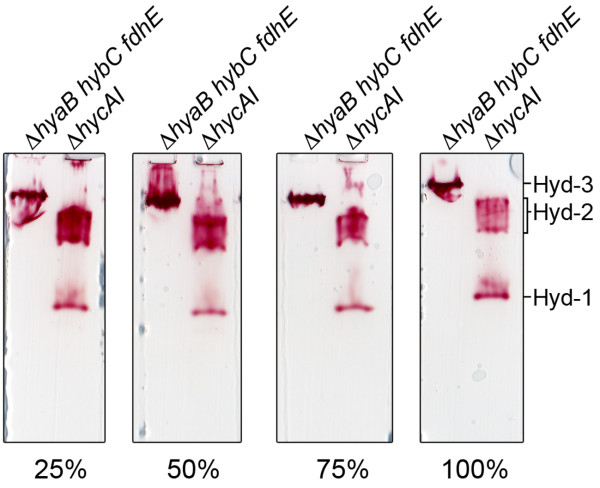
**Influence of hydrogen concentration on Hyd-3 activity.** Total cell extracts (25 μg of protein) from the strains CPD17 (Δ*hyaB hybC fdhE*) and CP971 (Δ*hycA-I*) after anaerobic growth in TGYEP, pH 6.5 were applied to native-PAGE (7.5% w/v polyacrylamide). The polypeptide complexes were separated and after prior incubation under 100% nitrogen, the respective volumes of pure hydrogen gas to deliver a final concentration of approximately 25%, 50%, 75% of pure hydrogen were added to the closed vessels and the pressure released. The 100% hydrogen atmosphere sample was stained under hydrogen flow until the bands appeared. The migration patterns of hydrogenase 1 (Hyd-1), Hyd-2 and Hyd-3 are given on the right hand side of the figure. Arrows indicate the top of the gel.

No Hyd-3 enzyme activity is detectable after non-denaturing PAGE if the hydrogen concentration in the gaseous phase approximates 5% (*ca*. 30-40 μM dissolved H_2_ at 1 atm. pressure and 25 °C [36]) or below (see Figure 1; [18,20]). To provide an estimate of the minimal H_2_ concentration in the gas headspace required to visualize Hyd-3 activity, we separated extracts derived from CP971 (Δ*hycA-I*) and CPD17 (Δ*hyaB hybC fdhE*) in native-PAGE and incubated these with different concentrations of H_2_ in the headspace (Figure 4). The results clearly show that from a concentration of 25% H_2_ in the gas phase (*ca*. 0.25 mM dissolved H_2_) Hyd-3 activity was detectable. The intensity of the Hyd-1 activity also remained comparatively constant at the different high hydrogen concentrations (Figure 4). In contrast, the intensity of the Hyd-2 activity bands decreased with increasing hydrogen gas concentration, suggesting an inverse correlation between Hyd-3 and Hyd-2 activities exists at high hydrogen gas concentration when BV is used as electron acceptor. We determined the redox potential (*E*_h_) of the BV/TTC assay buffer with 5% hydrogen in the headspace to be -264 mV and with 100% in the headspace to be -322 mV (Table 2).

**Table 2:** Redox potentials of the assay buffers

**Hydrogen in headspace**	**50 mM MOPS, pH 7**	**50 mM MOPS, pH 7, BV/TTC**^**a**^	**50 mM MOPS, pH 7, PMS/NBT**^**b**^	**50 mM MOPS, pH 7, NBT**
0%^c^	+ 170 mV	+ 78 mV	+ 74 mV	+ 73 mV
5%	- 120 mV	- 264 mV	- 38 mV	- 65 mV
100%	- 349 mV	- 322 mV	- 92 mV	- 102 mV

^**a**^ The concentrations of BV and TTC were 0.5 mM and 1.0 mM, respectively.

^b^ The concentrations of PMS and NBT were 0.3 mM and 0.2 mM, respectively.

^c^ Measured at 25 °C and 1 atm. pressure. 0% hydrogen indicates measurements were made in air. Note that all measurements were made twice.

### Hyd-1 catalyzes the hydrogen-dependent reduction of nitroblue tetrazolium

Through the analysis of extracts derived from anaerobically grown *E. coli* strains specifically unable to synthesize Hyd-1 (FTD22), Hyd-2 (FTD67), Hyd-3 (CP971), Hyd-1/Hyd-2 (CP734) or all three [NiFe]-hydrogenases (FTD147 and DHP-F2), it was shown that only strains able to synthesize Hyd-1 were capable of reducing nitroblue tetrazolium (NBT) in a hydrogen-dependent manner (Figure 2C, left panel). Notably, intensely stained activity bands of Hyd-1 were observed after only 5 min incubation with 5% H_2_ in the gas phase. The redox potential of the assay buffer in the presence of 5% headspace hydrogen was determined to be – 38 mV (Table 2), decreasing to – 98 mV with 100% hydrogen in the headspace. Hyd-2 was unable to reduce NBT even after an incubation period of 3 h, as only Hyd-1 was visualized for the wild-type MC4100 (Figure 2A). Incubation for 16 h did not alter this pattern of staining (data not shown). Equally, Hyd-3 was also incapable of transferring electrons to NBT (Figure 2C). Similarly, deletion of the genes coding for the putative Hyd-4 enzyme [37] in strain FTD150 also did not result in a different pattern from strain FTD147, which suggests that Hyd-4 is not active under the conditions tested.

To analyse the specificity of the apparent Hyd-1-dependent NBT stain, the strain FM460 (Δ*selC*) was employed and a crude extract derived from this strain displayed a Hyd-1 activity band of similar intensity to that in MC4100 but the extract lacked the slower migrating activity band confirming that this was due to Fdh-N and Fdh-O (Figure 2C, right panel), as previously reported [21]. A *selC* mutant is incapable of incorporating selenocysteine into proteins and so lacks all formate dehydrogenase activity [38]. Moreover, strains CPD17 and CPD23, both carrying a deletion in *fdhE*, and strain CPD24, which carries deletions in the genes encoding the large subunit of Fdh-N and Fdh-O (Figure 2C, right panel) also lacked the Fdh-N and Fdh-O activity bands, as anticipated. Taken together, the fast-migrating, H_2_-dependent NBT-reducing activity band shown here is not linked to formate dehydrogenase activity and is Hyd-1.

**Figure 5:**
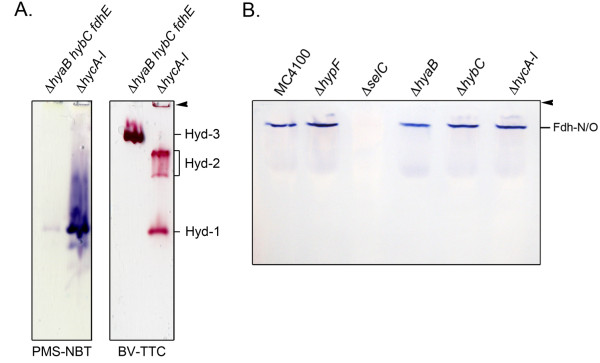
**Exclusive hydrogen-dependent reduction of nitroblue tetrazolium by Hyd-1 and the Fdh-N/O enzymes. A**: Total cell extracts (25 μg of protein) from the strains CPD17 (Δ*hyaB hybC fdhE*) and CP971 (Δ*hycA-I*) after anaerobic growth in TGYEP, pH 6.5 were applied to native-PAGE (7.5% w/v polyacrylamide) and the gels were subsequently stained for 3 h under a 100% hydrogen with PMS-NBT or BV-TTC as described in the Methods section. **B**: Cell extracts as in A from the strains MC4100, DHP-F2 (Δ*hypF*), FM460 (Δ*selC*), FTD22 (Δ*hyaB*), FTD67 (Δ*hybC*) and CP971 (Δ*hycA-I*) were submitted to native page (7.5% w/v polyacrylamide) and stained with PMS-NBT and formate under a 100% nitrogen atmosphere. The activities of the formate dehydrogenases N and O (Fdh-N/O) are given on the right hand side of the gel. Arrows indicate the top of the gel.

As a final control, we replaced the electron donor H_2_ with formate, the usual substrate of the formate dehydrogenases. The only activity detectable after native-PAGE and staining was that due to Fdh-N and Fdh-O (Figure 5B) and this activity was absent in extracts of strain FM460 (Δ*selC*).

### Reduction of NBT by Hyd-1 variants with amino acid exchanges in the supernumerary cysteines near the proximal [4Fe-3 S] cluster

**Figure 6:**
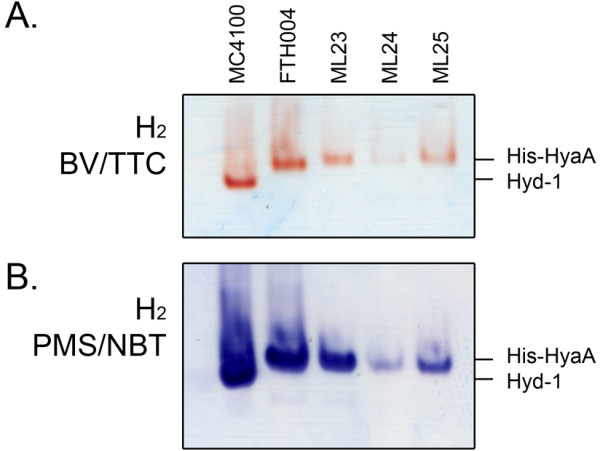
**Oxygen-sensitive variants of hydrogenase 1 catalyze hydrogen-dependent reduction of nitroblue tetrazolium.** The strains MC4100, its His-tagged HyaA derivative FTH004 and the respective HyaA cysteine exchange strains ML23 (C19G/C120G), ML24 (C120G) and ML25 (C19G) were grown anaerobically in TGYEP, pH 6.5 and 25 μg protein from crude extracts derived from the cells were loaded onto 7.5% (w/v polyacrylamide) non-denaturating-PAGE. Staining of the gels was performed as indicated on the left under a 100% hydrogen atmosphere in the presence of **A**: either BV and TTC or **B**: PMS and NBT as described in the Methods section. The migration pattern of the wild type hydrogenase 1 activity (Hyd-1) and the His-tagged form (His-HyaA) are marked on the right hand side.

Of the three hydrogenases synthesized in anaerobically growing *E. coli* cells only Hyd-1 can reduce NBT in a hydrogen-dependent manner. One of the major differences between Hyd-1 and the other enzymes is its oxygen tolerance [39]. The current proposed reason for the high oxygen tolerance exhibited by Hyd-1 is the unusual proximal [4Fe-3S]-cluster, along with two additional cysteinyl residues in the immediate environment around the cluster [9,40]. Indeed, recent site-specific mutagenesis experiments have identified Cys-19 as being particularly important for conferring oxygen-tolerance to the enzyme, because when substituted by glycine it generates an active Hyd-1 variant that is oxygen-sensitive [9]. In order to test whether the supernumerary cysteinyl residues (Cys-19 and Cys-120) are important for the ability of Hyd-1 to reduce NBT, we examined the H_2_-dependent NBT-reduction activity of extracts derived from strains encoding the HyaA small-subunit variants C19G and C120G variants of Hyd-1 [9]. All Hyd-1 variants present in crude extracts from anaerobically grown cells retained the ability to reduce both NBT and BV/TTC in the presence of hydrogen, indicating that the substitution of neither Cys-19 nor Cys-120 affects electron-transfer to the artificial electron acceptors (Figure 6).

### The core catalytic dimer of Hyd-1 reacts with NBT

**Figure 7:**
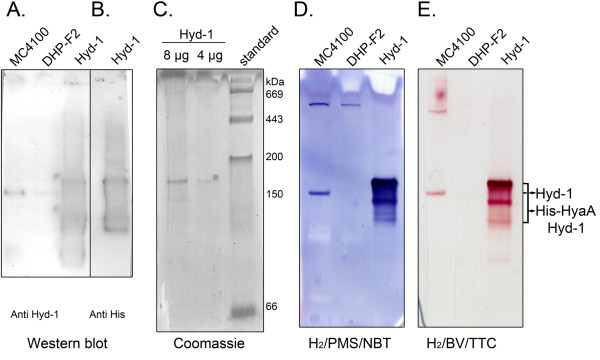
**The heterodimeric HyaB-His-HyaA complex of Hydrogenase 1 catalyzes the hydrogen-dependent reduction of NBT.** Aliquots of crude extracts (25 μg total protein) derived from strains MC4100 and DHP-F2 (Δ*hypF*) grown anaerobically in TGYEP, pH 6.5 or 8 μg of purified Hyd-1 from strain FTH004 were subjected to 7.5% (w/v polyacrylamide) non-denaturating PAGE and the gels were treated as follows: **A**. transferred to a nitrocellulose membrane and analyzed with antibodies directed against Hyd-1; **B**. transferred to a nitrocellulose membrane and analyzed with monoclonal His-tag antibody; **C**. the gel containing purified Hyd-1 and the molecular mass standard was stained with Coomassie Brilliant Blue. The masses of the standard proteins (Sigma) are given on the right hand of the panel. Alternatively, the extracts and purified enzyme were: **D**. stained for 10 minutes under a 100% hydrogen atmosphere with PMS and NBT as electron acceptors; or **E**. stained under a hydrogen atmosphere with BV and TTC as electron acceptors. The bands assigned to Hyd-1 activity or the His tagged version of HyaA-Hyd-1 activity are indicated on the right hand of the gels.

Recent studies have shown that the small subunit of the *E. coli* hydrogenases must form a complex with the large subunit for electron transfer from hydrogen to BV to occur [20,41]. Although not yet unequivocally demonstrated, it is conceivable that the artificial electron acceptors BV and NBT receive electrons directly from one of the [Fe-S]-clusters in the HyaA small subunit of Hyd-1. The HyaA small subunit of the core catalytic HyaAB dimer of Hyd-1, when correctly assembled in the membrane, conducts electrons through a [Fe-S]-cluster relay between the active site within the large subunit and a proximal *b*-type heme located within a membrane-integral cytochrome *b* subunit (HyaC). This is different for Hyd-2, because there is no HyaC equivalent and instead the small subunit HybO interacts with an additional [Fe-S] cluster-containing subunit, HybA, and the HybB integral membrane protein [34,42]. It is possible, therefore, that NBT receives electrons from the cytochrome *b* subunit HyaC and not from HyaA. To test this a hexa-histidine affinity tagged variant of Hyd-1 [34] was isolated from the membrane fraction of anaerobically grown FTH004. Since the HyaC subunit is only loosely bound to Hyd-1 in detergent, this allows the isolation of the active, core heterodimer comprising HyaB and HyaA. The authenticity of the purified His-tagged Hyd-1 enzyme was verified by Western blot detection using anti-Hyd-1 antibodies (Figure 7A and B) and the quality of the purified enzyme was analysed by Coomassie Brilliant Blue staining (Figure 7C). Native electrophoresis followed by activity staining with hydrogen and NBT revealed that the core heterodimer retained both NBT- (Figure 7D) and BV/TTC-reducing (Figure 7E) activities after native-PAGE. Therefore, it can be concluded that membrane-anchoring subunit HyaC is not required for electron-transfer to NBT.

## Discussion

Tetrazolium-based redox dyes are useful tools in zymographic detection of oxidoreductase enzyme activity in non-denaturing PAGE because upon irreversible reduction they generate coloured, insoluble formazan complexes, which are advantageous in cumulative staining procedures. Triphenyl tetrazolium has been used for a considerable time as a means of distinguishing the hydrogenase enzymes in *E. coli* cell extracts [18,19]. Measuring Hyd-3 activity in the presence of the H_2_-oxidizing enzymes was problematic in the past and visualizing it had not been successfully accomplished until the current study was conducted. However, optimization of the in-gel assay conditions, together with the judicious use of defined mutants has allowed us for the first time to visualize Hyd-3 activity unequivocally after native-PAGE. The complexes exhibiting Hyd-3 activity migrate in native-PAGE at high molecular masses, similar to the trimer of trimers of the Fdh-N and Fdh-O with a mass of 500-550 kDa [21]. This suggests that the stoichiometry of the individual components in the FHL complex might be greater than unity. Nothing is currently known about the stoichiometry of the FHL complex components or the architecture of the HycE/HycG large and small subunit within the complex, and this will form the subject of future studies.

The findings of the current study suggest that while the Fdh-H component of the FHL complex is required for maximal activity of the complex, in its absence activity of the Hyd-3 can still be detected and its migration position in the gel system is very similar in extracts of the wild-type and the *fdhF* mutant. This suggests perhaps that the Fdh-H component is separated from the rest of the complex during electrophoresis. The lability of the Fdh-H activity has been noted previously [15,43].

One possible reason why the Hyd-3 activity was previously overlooked after in-gel staining is the considerable overlap in the staining pattern of Fdh-N/O, Hyd-3 and Hyd-2. Alternatively, reliable detection of Hyd-3 activity appears to require hydrogen concentrations of minimally 5% in the gas phase and many of our previous studies used lower concentrations [20]. Using high concentrations of hydrogen in the staining procedure has the advantage that Hyd-3 activity is detectable after a few minutes’ exposure, while Hyd-2 is not detectable under these conditions, possibly due to the low abundance of the enzyme in extracts of *E. coli* coupled with the brief exposure to hydrogen. Hyd-3, like Hyd-1, is a more abundant enzyme and this possibly explains the rapid visualization of both these enzymes after only 10 min exposure to high hydrogen concentrations.

The fact that the FHL complex is active in H_2_ oxidation contrasts the physiological direction of the reaction in the *E. coli* cell. This, therefore, might be an explanation for the comparatively high H_2_ concentrations required to drive the reaction in the direction of hydrogen oxidation. The similar redox potentials of formate and hydrogen do, however, indicate that this reaction should be freely reversible, possibly pointing to a role of a progenitor of the FHL complex in CO_2_ fixation [44].

Another possible explanation for the effect of hydrogen concentration on Hyd-3 activity is that high hydrogen concentrations drive the redox potential of a solution to more negative *E*_h_ values [10]. For example a 100% hydrogen atmosphere will result in a *E*_h_ = -420 mV in anaerobic cultures, while a 5% hydrogen concentration in the headspace equates to a redox potential of around -370 mV and a dissolved hydrogen concentration in cultures of maximally 40 μM at 25°C [36].

Our recent studies have shown that the [Fe-S]-cluster-containing small subunit of the hydrogenase must be associated with the large subunit in order for hydrogen-dependent BV reduction to occur [20]. It is possible that BV receives electrons from a [Fe-S] cluster. If this is the case, then hydrogen-dependent BV reduction by a component of Hyd-3 also possibly occurs via a [Fe-S] cluster; however, due to the considerable number of [Fe-S] cluster-containing subunits in the complex (HycB, HycF, HycG and the Fdh-H enzyme itself [20,45]) future studies will be required to elucidate whether BV can interact with one or several sites in the complex.

The use of the electron acceptor NBT enabled a clear distinction between Hyd-1 and Hyd-2 activities. Previous experiments have shown that PMS/NBT staining is sometimes non-specific due to interaction with protein-bound sulfhydryl groups and even BSA was shown to be capable of staining gels incubated with PMS/NBT [46]. We could clearly show in this study, however, that, of the hydrogenases in *E. coli*, only Hyd-1 was capable of the specific, hydrogen-dependent reduction of PMS/NBT. Notably, both respiratory Fdhs also showed a strong NBT-reducing activity, which correlates well with previous findings for these enzymes [21].

Hyd-1 is similar to the oxygen-tolerant hydrogenases of *R. eutropha* and it is equipped with two supernumerary cysteinyl residues, which coordinate the proximal [4Fe-3S]-cluster [9,47]. PMS-mediated staining has been previously used for the oxygen-tolerant hydrogenases from *R. eutropha*[22,23], which led to the suggestion that particular structural features of oxygen-tolerant hydrogenases accounted for the differences in dye-reducing activity of the oxygen-tolerant and sensitive enzymes. The supernumerary Cys-19 of the small subunit, when exchanged for a glycine was shown to convert Hyd-1 from an oxygen-tolerant to an oxygen-sensitive enzyme [9]. This amino acid exchange did not affect NBT reduction in our assay system, thus indicating that the oxygen-tolerance is not the sole reason for the ability of Hyd-1 to reduce NBT. This finding is also in agreement with the recent observation that the exchange of the supernumerary cysteines does not affect the catalytic bias of Hyd-1 to function in hydrogen-oxidation [9]. The structural and electronic properties of Hyd-1 [40] probably govern its ability to transfer electrons from hydrogen to comparatively high-potential redox dyes such as NBT (*E*_h_ value of -80 mV). The similar redox potential of NBT in our assay buffer with and without PMS (see Table 2), indicates that Hyd-1 should reduce NBT directly, which is indeed what we have observed (data not shown).

Neither Hyd-3 nor Hyd-2 can reduce NBT and this is presumably because they function optimally at very low redox potentials, although potential steric effects restricting interaction of the enzymes with the dye cannot be totally excluded at this stage. Hyd-2 is a classical hydrogen-oxidizing enzyme that functions optimally at redox potentials lower than -100 to -150 mV [8,10]. The combined inclusion of BV (*E*_h_ = -360 mV) and TTC (*E*_h_ = -80 mV), along with 5% hydrogen in the headspace, of the assay was sufficient to maintain a low redox potential to detect Hyd-2 readily. This also explains why long incubation times are required for visualization of Hyd-1 activity with the BV/TTC assay. Increasing the hydrogen concentration in the assay to 100% drives the redox potential below -320 mV and explains why the Hyd-3 activity was readily detectable at hydrogen concentrations above 25% (see Figure 4).

In stark contrast to Hyd-2 and Hyd-3, Hyd-1 shows a high activity at redox potentials above -100 mV [8,10]. In the assay system used in this study, the presence of NBT in the buffer system resulted in a redox potential of -65 mV in the presence 5% hydrogen and -92 mV when the hydrogen concentration was 100%, both of which are optimal for Hyd-1 activity and well above that where the Hyd-2 is enzymically active [8,10]. Placed in a cellular context, this agrees perfectly with the roles of Hyd-2 in coupling hydrogen oxidation to fumarate reduction, of Hyd-1 in scavenging hydrogen during microaerobiosis and of Hyd-3 in functioning at very low redox potentials in proton reduction [1]. This allows the bacterium to conduct its hydrogen metabolism over a very broad range of redox potentials.

## Conclusions

Using increased partial pressure of dihydrogen in combination with the artificial electron acceptor combination benzyl viologen/triphenyl tetrazolium chloride, we defined conditions allowing the identification of an active Hyd-3 enzyme complex after non-denaturing gel electrophoresis. Moreover, by substituting BV/TTC with nitroblue tetrazolium as an electron acceptor we could demonstrate that only the oxygen-tolerant Hyd-1 enzyme could catalyse hydrogen-dependent dye reduction, suggesting that this facile assay could be used to identify oxygen-tolerant hydrogenases in other microorganisms. However, the ability of Hyd-1 to reduce NBT was not dependent on the oxygen-tolerance of the enzyme because an oxygen-sensitive Hyd-1 variant in which the supernumerary Cys-19 was substituted by Gly retained the ability to reduce the redox dye.

## Methods

### Strains and growth conditions

All strains used in this study are listed in Table 1. *E. coli* strains were routinely grown at 37°C on LB-agar plates or with shaking in LB-broth [48]. Plates were solidified by adding 1.5% (w/v) agar to the media. Anaerobic growths were performed at 37°C as standing liquid cultures. Cultures for determination of enzyme activity were grown in TGYEP media [49] containing 1% (w/v) peptone, 0.5% (w/v) yeast extract, 0.1 M potassium buffer pH 6.5 and the cultures were supplemented with 0.8% (w/v) of glucose. When required, the antibiotics kanamycin and chloramphenicol were added to the culture media to the final concentration of 50 μg and 12 μg per ml, respectively. The strains CPD17, CPD23 and CPD24 were constructed using P1*kc* phage transduction to move the respective defined deletion mutation from the appropriate strains obtained from the Keio collection [48,50]. When required the plasmid pCP20 was used to remove the antibiotic resistance cassette as described [51].

#### Polyacrylamide gel electrophoresis

Non-denaturing PAGE was performed using a discontinuous system with 7.5% (w/v) polyacrylamide separating gels in 250 mM Tris/HCl buffer, pH 8.5 including 0.1% (w/v) Triton X-100 [18]. As running buffer 0.1 M Tris/0.1 M glycine buffer was used. After reaching mid-exponential phase of growth cells were harvested from cultures by centrifugation at 10,000 x g for 15 min at 4 °C and after washing once in the same volume of 50 mM MOPS buffer pH 7.0, cells were resuspended in a tenth of their volume of 50 mM MOPS buffer pH 7.0, broken by sonification and cell debris and unbroken cells removed as described [20]. Samples of crude extract were resuspended at a protein concentration of 10 mg ml^-1^ in 50 mM MOPS buffer pH 7.0 and incubated with a final concentration of 5% (w/v) Triton X-100 prior to application of the solubilized sample (usually 25 μg of protein) to the gels. Alternatively, for neutral pH analyses the barbitone gel system was used. This system uses final concentrations of 34 mM Tris-phosphate buffered stacking gel, pH 5.5 and 62.5 mM Tris-HCl resolving gel pH 7.5. The running buffer consists of 82.5 mM Tris and 26.8 mM diethylbarbituric acid, pH 7.0. Hydrogenase activity-staining was done as described in [18] with 0.5 mM benzyl viologen (BV) and 1 mM 2,3,5,-triphenyltetrazolium chloride (TTC) and continuous flushing with highly pure hydrogen gas until the activity bands appeared except that the buffer used was 50 mM MOPS pH 7.0. Alternatively, staining was done in hydrogen-flushed buffer using 0.3 mM phenazine methosulfate (PMS) as mediator and 0.2 mM nitroblue tetrazolium (NBT) as electron acceptor [52]. When formate was added as substrate to the buffer, a final concentration of 50 mM was used. When used in native-PAGE molecular mass standard proteins from a gel filtration markers kit 29-700 kDa (Sigma) were mixed in equal amounts and 6 μg of each were loaded on the gel.

#### Immunological and enzymic methods

Western blotting was performed as described in [53] by transferring proteins to nitrocellulose membranes and challenging them with monoclonal penta-His antibody from mouse (Qiagen) or polyclonal anti-Hyd-1 antibody (1:20000). Secondary goat-anti-mouse or anti-rabbit antibody, respectively conjugated with HRP enzyme (Bio-Rad, USA) was used for visualisation with the Immobilon Western chemiluminescent HRP substrate (Millipore, USA). Purification of active Hyd-1 from a 5 L culture of strain FTH004 (His-HyaA) grown in TGYEP, pH 6.5 supplemented with 5 μM Ni^2+^ was carried out as described [34]. Determination of protein concentration was done by the method of Bradford (Bio-Rad, USA) [54].

#### Measurement of redox potential

Aliquots of 50 mM MOPS buffer pH 7.0 containing the concentrations of the respective redox dyes indicated above were either incubated overnight in an anaerobic chamber with an atmosphere containing 5% hydrogen for 6 h or was bubbled with hydrogen gas (100% atmosphere) for 30 min and the redox potential determined using a EMC 30-K010-D redox micro-electrode (Sensortechnik Meinsburg GmbH, Germany) attached to a Lab850 pH/redox meter (Schott Instruments, Germany). The electrode was standardized using a redox buffer provided by the company. Measurements were performed two times.

## Abbreviations

FHL, Formate hydrogenlyase; Hyd, Hydrogenase; Fdh, Formate dehydrogenase; BV, Benzyl viologen; TTC, 2,3,5-triphenyltetrazolium chloride; NBT, Nitroblue tetrazolium; PMS, Phenazine methosulfate.

## Competing interests

The authors declare that they have no competing interests.

## Authors’ contributions

CP carried out the experimental studies and drafted the manuscript. MJ conducted the redox potential measurements and the gel staining experiments, RGS and FS conceived and coordinated the study and drafted the manuscript. All authors read and approved the final manuscript.
